# Differential transmission of the molecular signature of RBSP3, LIMD1 and CDC25A in basal/ parabasal versus spinous of normal epithelium during head and neck tumorigenesis: A mechanistic study

**DOI:** 10.1371/journal.pone.0195937

**Published:** 2018-04-19

**Authors:** Shreya Sarkar, Neyaz Alam, Syam Sundar Mandal, Kabita Chatterjee, Supratim Ghosh, Susanta Roychoudhury, Chinmay Kumar Panda

**Affiliations:** 1 Department of Oncogene Regulation, Chittaranjan National Cancer Institute, Kolkata, India; 2 Department of Surgical Oncology, Chittaranjan National Cancer Institute, Kolkata, India; 3 Department of Epidemiology and Biostatistics, Chittaranjan National Cancer Institute, Kolkata, India; 4 Department of Oral and Maxillofacial Pathology, Buddha Institute of Dental Sciences and Hospital, Patna, India; 5 Department of Oral and Maxillofacial Pathology, Burdwan Dental College and Hospital, Burdwan, India; 6 Basic Research, Saroj Gupta Cancer Centre and Research Institute, Kolkata, India; University of Navarra, SPAIN

## Abstract

Head and neck squamous cell carcinoma (HNSCC) is a global disease and mortality burden, necessitating the elucidation of its molecular progression for effective disease management. The study aims to understand the molecular profile of three candidate cell cycle regulatory genes, RBSP3, LIMD1 and CDC25A in the basal/ parabasal versus spinous layer of normal oral epithelium and during head and neck tumorigenesis. Immunohistochemical expression and promoter methylation was used to determine the molecular signature in normal oral epithelium. The mechanism of alteration transmission of this profile during tumorigenesis was then explored through additional deletion and mutation in HPV/ tobacco etiological groups, followed byclinico-pathological correlation. In basal/parabasal layer, the molecular signature of the genes was low protein expression/ high promoter methylation of RBSP3, high expression/ low methylation of LIMD1 and high expression of CDC25A. Dysplastic epithelium maintained the signature of RBSP3 through high methylation/ additional deletion with loss of the signatures of LIMD1 and CDC25A via deletion/ additional methylation. Similarly, maintenance and / or loss of signature in invasive tumors was by recurrent deletion/ methylation. Thus, differential patterns of alteration of the genes might be pre-requisite for the development of dysplastic and invasive lesions. Etiological factors played a key role in promoting genetic alterations and determining prognosis. Tobacco negative HNSCC patients had significantly lower alterations of LIMD1 and CDC25A, along with better survival among tobacco negative/ HPV positive patients. Our data suggests the necessity for perturbation of normal molecular profile of RBSP3, LIMD1 and CDC25A in conjunction with etiological factors for head and neck tumorigenesis, implying their diagnostic and prognostic significance.

## Introduction

An omnipresent acrimony, Head and Neck Squamous Cell Carcinoma (HNSCC),comprising of oral, nasopharyngeal and laryngeal cancers represents> 95% of all head and neck (H&N) malignancies [1]. It presents sixth highest global prevalence, constituting30–40% of total cancer incidents in the Indian subcontinent, with tobacco, betel quid, alcohol and Human Papilloma Virus (HPV) as risk factors [2].Evidence indicate the pre-requisition of non- random aberrations (epigenetic/ genetic) in the normal basal stem-like layer for epithelial tumorigenesis including HNSCC [3, 4],although the molecular events involved are elusive. Thus, understanding the molecular mechanism of HNSCC development entails an analysis of the profile of candidate genes in the normal basal/parabasal epithelium, followed by their alterations in different stages of tumorigenesis.

Loss of heterozygosity in chromosomal 3p21.2–22 region in HNSCC patients in India were identified previously [5], along with several candidate tumor suppressor genes (TSGs) including RBSP3 and LIMD1, which were associated with mild dysplasia and CDC25A, which was associated with moderate dysplasia of H&N [6, 7].

Subsequently, in a preliminary study, immunohistochemical examination revealed association of LIMD1 with chronic ulceration and RBSP3 with hyperplasia of H&N with distinct patterns of expression in its normal epithelium [8, 9].Although the mechanisms behind their disparate expression in normal epithelium is ambiguous, differential epigenetic alterations, particularly promoter hypermethylation, might be important in regulating gene expression in the normal epithelium due to its decisive role in cellular differentiation [10, 11]. To the best of our knowledge, the changes in promoter methylation of the candidate genes in different layers of normal epithelium are yet to be unraveled. Moreover, which signature, the basal/ parabasal, or that of the spinous layer in normal epithelium gets transmitted during progressive development of HNSCC, or how it alters in tumorigenesis, along with the role of etiological factors, especially tobacco and HPV is yet to be understood. Thus, analysis of the status of RBSP3, LIMD1 and CDC25A in basal/parabasal versus spinous of normal oral epithelium, followed by their alterations (methylation/ deletion/ mutation/ expression) are essential to interpret their role during tumorigenesis.

In this study, immunohistochemical expression profile (IHC) of the proteins was first evaluated in different layers of normal oral epithelium, dysplastic oral epithelium and HNSCC, followed by correlation with respective promoter methylation patterns. The incidence of genetic alterations was then evaluated at different clinical stages, along with clinico-pathological correlation to determine possible associations, in the same set of samples. Our data indicates that compared to normal epithelium, low basal/ parabasal expression of RBSP3 was maintained, while the high expression of LIMD1 and CDC25A were lost during tumour development due to genetic/ epigenetic alterations. Etiological factors, especially HPV played an important role in patient prognosis, thus indicating the utility of the genes as important diagnostic/ prognostic markers in HNSCC.

## Materials and methods

### Study population and H&N lesions

**Fig 1:**
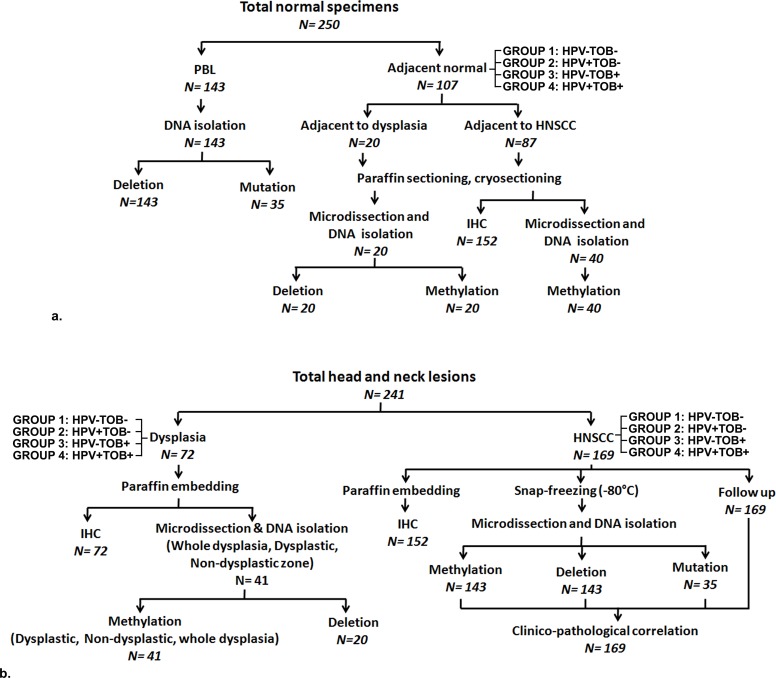
Experimental work flow and sample usage. Schematic diagram representing of type, distribution and usage of samples in different experimental procedures and etiological groups. *N*: Number of samples; PBL: Peripheral blood lymphocytes; HNSCC: Head and neck squamous cell carcinoma; IHC: Immunohistochemistry. HPV: Human papilloma virus; TOB: Tobacco. a. Normal specimens. b. Head and neck lesions.

H&N lesions and paired adjacent normal epithelium/blood were collected from unrelated patients (Dysplasia = 72, HNSCC = 173) from the Hospital Section of Chittaranjan National Cancer Institute, Kolkata with their written informed consent and approval from the Institutional Ethical Committee of Chittaranjan National Cancer Institute, Kolkata, India and hospital authorities. A part was immediately stored at -80°C, while the other was kept in formalin (Fig 1). Tumors were graded/staged as per UICC TNM classification [12] by two independent pathologists. Simultaneously considering two important etiological factors HPV infection and tobacco habit (TOB), tumors were divided into four groups: HPV-TOB- (Group 1), HPV+TOB- (Group 2), HPV-TOB+ (Group 3) and HPV+TOB+ (Group 4) (Fig 1, Table 1).

**Table 1:** Clinico-pathological features of pre-malignant (dysplasia) and invasive (squamous cell carcinoma) head and neck lesions. Abbreviations: n: Number of samples; BM: Buccal mucosa; ALV: Alveolus; TON: Tongue -ve: Factor absent; +ve: Factor present. p value represents level of significance during comparison.

Clinical	PRE-MALIGNANT					MALIGNANT				
Features			HPV 16/18 Positivity		p VALUE			HPV 16/18 Positivity		p VALUE
	N	%	+ve (n%)	-ve (n%)		N	%	+ve (n%)	-ve (n%)	
	72		38 (52.78)	34 (47.22)	0.61	173		108 (62.43)	65 (37.57)	**<0.001**
**Age**										
**Mean+- SD**	54 ± 13.42				50 ± 12.44			
Mean ≤	46	63.89	26 (56.52)	20 (43.48)	0.46	71	41.04	40 (56.33)	31 (43.67)	0.2
Mean >	26	36.11	12 (46.15)	14 (53.85)		103	58.96	68 (66.02)	34 (33.98)	
**Primary site**										
BM	47	65.28	26 (55.32)	21 (44.68)	0.93	83	47.97	54 (65.06)	29 (34.94)	0.66
ALV	9	12.5	3 (33.33)	6 (66.67)		32	18.5	18 (56.25)	14 (43.75)	
TON	16	22.22	10 (62.5)	6 (37.5)		22	12.72	14 (63.64)	8 (36.36)	
Others	-	-	-	-		36	20.81	22 (61.11)	14 (38.89)	
**Gender**										
Male	53	73.61	30 (57.14)	23 (42.86)	0.79	128	74.00	79 (61.72)	49 (38.28)	0.85
Female	19	26.39	10 (52.63)	9 (47.63)		45	26.00	29 (64.44)	16 (35.56)	
**TMN Stage**	N.A.									
I						15	8.67	10 (66.67)	5 (33.33)	1
II						31	17.92	19 (61.29)	12 (38.71)	
III						86	49.71	52 (60.47)	34 (39.53)	
IV						41	23.7	27 (65.85)	14 (34.15)	
**Grade**										
Mild/ I	32	44.45	15 (46.88)	17 (53.12)	0.31	43	25.29	27 (61.36)	16 (38.64)	0.93
Moderate/ II	16	22.22	8 (50)	8 (50)		98	56.32	60 (61.22)	38 (38.78)	
Severe/ III	24	33.33	15 (62.5)	9 (37.5)		32	18.39	21 (65.63)	11 (34.37)	
**Lymph Node**	N.A.									
+ve						88	50.87	56 (63.64)	31 (36.36)	0.64
-ve						85	49.13	52 (61.18)	34 (40.48)	
**Tobacco**										
+ve	52	72.22	28 (53.85)	24 (46.15)	0.78	125	72.25	81 (64.8)	44 (35.2)	0.3
-ve	20	27.78	10 (50)	10 (50)		48	27.25	27 (56.25)	21 (43.75)	
**Tumor groups**										
**1**	9	12.5	0 (0)	9 (100)		11	7.64	0 (0)	11 (100)	
**2**	11	15.28	11 (100)	0 (0)		33	22.92	33 (100)	0 (0)	
**3**	25	34.72	0 (0)	25 (100)		37	25.69	0 (0)	37 (100)	
**4**	27	37.5	27 (100)	0 (0)		63	43.75	63 (100)	0 (0)	

### Microdissection and genomic DNA isolation

These were performed in two different sets:

#### 1. Separately from basal/parabasal and spinous layers of normal oral epithelium adjacent to tumours

**Fig 2:**
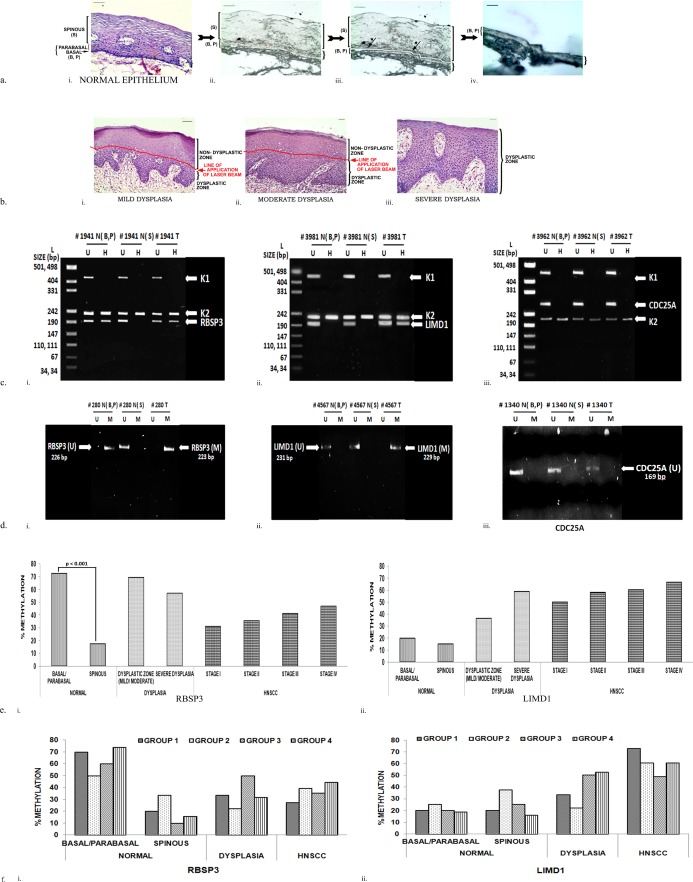
Promoter methylation analysis. Promoter methylation of the genes in normal epithelium (basal/ parabasal and spinous layers), dysplastic zone of epithelium and HNSCC. U: Unmethylated; M: Methylated; N: Normal; T: Tumor; B: Basal layer; P: Parabasal layer; S: Spinous layers; L: pUC19/ HpaII molecular weight ladder. a. Representative images depicting step- wise separation of basal/parabasal and spinous layers in normal epithelium.i. Hematoxylin and eosin stained parallel normal section. ii. Unstained parallel section. iii. Laser line (arrow star) demarcating basal/ parabasal and spinous layers. iv. Remaining basal/ parabasal layers after separation of the spinous layers. b. Representative hematoxylin and eosin stained sections for separation of dysplastic and non- dysplastic zone in mild/ moderate and severe dysplasia. i. Mild dysplasia; ii. Moderate dysplasia; iii. Severe dysplasia. Red line represents the line of application of laser beam for separating dysplastic and non- dysplastic zones. c. Representation agarose gel image showing methylation by Methylation Specific Restriction Analysis (MSRA). U: Undigested; H: HpaII digested. d. Representation agarose gel image showing methylation by Methylation Specific PCR (MSP). U: Unmethylated; M: Methylated. e. Histograms depicting percentage of methylation obtained for i. RBSP3; ii. LIMD1. f. Histograms depicting percentage of methylation in etiological groups in i. RBSP3; ii. LIMD1. p value (Fisher’s exact) represents the level of significance during univariate comparison. # Represents sample number; B: Basal; P: Parabasal: S: Spinous: T: Tumor; N: Normal. Group 1: HPV-TOB-; Group 2: HPV+TOB-; Group 3: HPV-TOB+; Group 3: HPV+TOB+.

Basal/parabasal and spinous layers were identified in 5μm Hematoxylin and Eosin (H&E) stained serial paraffin/cryosections of normal oral epithelium adjacent to tumours (N = 40). After comparing the stained sections with unstained parallel tissue sections, layers were separated by Laser Capture Microdissection (LCM) procedure using PalmRobo software (Palm Microbeam, Zeiss, Germany) [13]. After demarcating basal/parabasal and spinous layers using a laser line, individual layers were laser-catapulted into separate microfuge tubes (Figs 1A and 2). DNA from each layer was then separately extracted by standard procedure using Proteinase K digestion, followed by phenol- chloroform extraction [10].

#### 2. H&N lesions and blood

The 5μm H&E sections from H&N lesions were identified for dysplasia (N = 41) and tumor- rich regions (N = 143), enriched (>60% lesions) by microdissection under a dissecting microscope (Leica MZ16, Germany) and LCM for removing contaminating normal cells (Fig 1B, Fig 2A and 2B).Genomic DNA from these and blood were isolated by the previously mentioned procedure.

### Immunohistochemical characterization

Expression of LIMD1, RBSP3 and CDC25A were studied by immunohistochemistry [12] in normal oral epithelium adjacent to tumours (N = 87), dysplastic oral epithelium (N = 72) and HNSCC (N = 152) in etiological groups 1–4 (Fig 1).Primary antibodies LIMD1 (CP-30-09) and RBSP3 (CP-57-09) were custom made by Imgenix India Pvt. Ltd. and previously validated [8], whileCDC25A (sc-6947), secondary antibodies rabbit anti-goat (sc-2020) and goat anti-rabbit (sc-2004) HRP conjugated secondary antibodies were from Santa Cruz Biotechnology, CA, USA. Mouse monoclonal LIMD1 obtained as a kind gift from Dr. Tyson Sharp, Barts Cancer Institute, London, UK. Primary antibodies (1:100), secondary antibodies (1:500), 3, 3’ diaminobenzidine (DAB)as the chromogen and hematoxylin as the counter stain were used and slides were photographed under a Bright Field microscope (Leica DM1000, Germany). Positive cells (<1% = 0, 1–20% = 1, 20–50% = 2, 50–80% = 3 and >80% = 4) and staining intensity (1 = weak, 2 = moderate, 3 = strong) was checked by two independent observers and combined to evaluate the ultimate expression (0–2 = low, 3–4 = intermediate, 5–7 = high) [14].

### Promoter methylation profiling

Promoter methylation was performed by PCR based Methylation Sensitive Restriction Analysis (MSRA)using HpaII restriction enzymes (Roche, Switzerland) in normal basal/parabasal and spinous layers of oral epithelium (N = 40), dysplastic oral epithelium (N = 41) and HNSCC (N = 143) in etiological groups 1–4. Results were validated using Methylation-Specific PCR (MSP) in normal oral epithelium (N = 10), dysplasia (N = 8) and HNSCC (N = 32) according to standard protocol [15] (primer details in S1 Table).

### Deletion analysis

Deletion with informative and non-informative microsatellite markers was studied in dysplastic (N = 20) and HNSCC samples (N = 143) in Groups 1–4. In deletion mapping using microsatellites, a standard polymerase chain reaction was performed with [γ-P32] dATP end- labeled forward primer in a 20 μl reaction mixture. For non- informative markers, analysis was performed using SST as a control locus, followed by electrophoresis in a denaturing acrylamide gel and autoradiography, as described previously [6, 7] (primers in S2 Table).

### Mutation detection

Mutations were screened in the functional domains, viz., RB binding LIM domain of LIMD1 (Exon 1) and phosphatase domains of RBSP3 (Exon 6–8) and CDC25A (Exon 7, 10–12) (S1 Table) by Single Strand Conformation Polymorphism (SSCP). In brief, in a standard PCR was performed using radiolabeled α- P^32^- dCTP, followed by electrophoresis in a non- denaturing acrylamide gel and analysis. Results were validated by sequencing using 3130xl Genetic Analyzer (Applied Biosystems, USA) in 35 paired normal/ HNSCC [16].

### Detection of HPV 16 and 18

HPV infection was detected by PCR using primers (MY09/MY11) designed from the consensus L1 region of the virus, followed by typing positive samples for HPV16/18 using type specific primers designed from the E6 region of HPV 16 and LCR region of HPV 18. Results were validated by Southern hybridization using detection and type specific probes [17].

### Clinico-pathological correlation

Risk associated with alterations and clinico-pathological factors was calculated using univariate (Fischer’s exact test determining Odds ratio) and multivariate (Multivariate Cox proportional hazard regression model determining Hazard Ratio(HR)) analysis. Chi square (trend) was used to associate alterations with increasing tumor stages/grades. Kaplan- Meir method was used to plot survival curves to assess relationship between 5-year disease free survivals with/without alterations. Log rank test evaluated differences in survival between different parameters. All statistical tests were two-sided with probability (p) value <0.05 considered significant. All calculations were by softwares Epi Info 7 (CDC, Atlanta) and IBM SPSS 21 (SPSS, Chicago, IL).

## Results

### Demography of the patients

Patients presenting with head and neck lesions were predominantly male, with a median age of 54 for dysplastic lesions and 50 for HNSCC and with buccal mucosa as the most common site affected (Table 1). Most patients were habitual users of tobacco.

### Differential distribution of patients in tumour groups according to tobacco habit and HPV infection

Presence of HPV increased from dysplastic lesions (52.78%) to malignant tumours (62.43%, Table 1), the most common subtype being HPV 16 (data not shown).

Moreover, by simultaneously considering two important etiological factors, tobacco usage (T) and HPV infection (H), patients were segregated into four Etiological Groups (1–4). By doing this, a disparate distribution of patients was observed with a higher fraction of patients being positive for both etiological factors tobacco and HPV (Group 4: dysplasia, 37.5%; HNSCC, 43.75%) (Table 1).

### Candidate genes show differential patterns of expression in basal/ parabasal versus spinous layers of normal head and neck epithelium but show similar low expression in head and neck lesions

**Fig 3:**
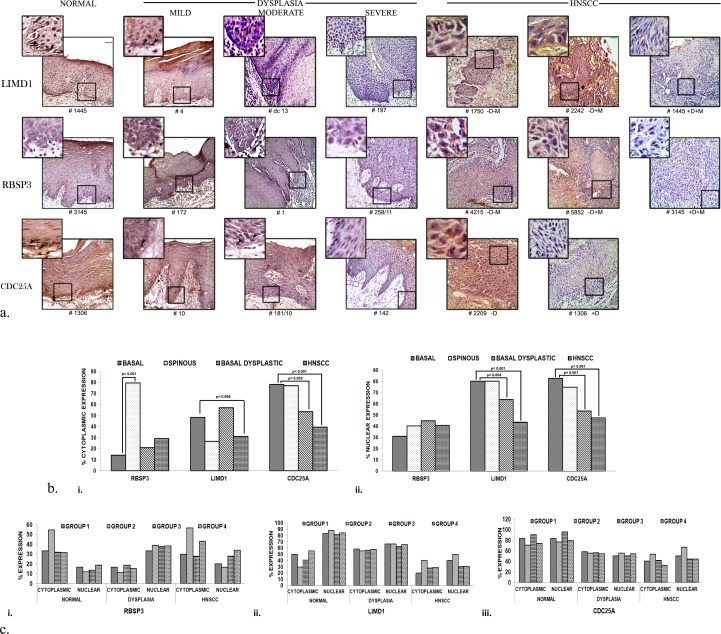
Expression profiling by of genes by immunohistochemistry (IHC). Representative immunohistochemical expression images and histograms in normal epithelium (basal, spinous layers), dysplasia and HNSCC, and in different etiological groups. a. Expression of the proteins during tumor progression from normal oral epithelium. RBSP3 showed low expression in the basal layer with high cytoplasmic expression in the spinous layers. LIMD1 and CDC25A exhibited high nuclear and high/moderate cytoplasmic expression throughout the normal epithelium. Expression of proteins gradually decreased during tumor progression. b. i. Percentage of cytoplasmic expression. ii. Percentage of nuclear expression. c. Percentage of expression in etiological groups. i. RBSP3 ii. LIMD1 iii. CDC25A p value (Fisher’s exact) represents the level of significance during comparison. Group 1: HPV-TOB-; Group 2: HPV+TOB-; Group 3: HPV-TOB+; Group 3: HPV+TOB+ Arrowheads represent nuclear expression. Scale bar: 50μm. Outer magnification: 20X, inset 40X. #: Sample number.

RBSP3 showed low cytoplasmic/nuclear expression in the basal/parabasal layer of normal oral epithelium (14%, 31% cases respectively) with significant increased cytoplasmic expression in spinous layer (79%) (Fig 3A and 3B). Like basal/parabasal layer of normal oral epithelium, comparable frequencies of cytoplasmic/nuclear expression were obtained in dysplastic lesions (21%, 44% respectively) and HNSCC (29%, 41% respectively) (Fig 3A and 3B). In contrast, LIMD1 and CDC25A showed high nuclear expressions in both basal/parabasal (80.06%, 82.76% respectively) and spinous layers (80.06%, 74.71%) of normal epithelium with significant loss of their expression during development of dysplasia (63.88%, 52.78%) and HNSCC (43.42%, 47.37%) (Fig 3A and 3B).

Comparing the patterns of their expression in etiological Groups 1–4, similar expression of all genes was observed in normal epithelium and during tumorigenesis (Fig 3C), indicating that etiological factors might not play a role in determining expression of the proteins.

Therefore, low expression of RBSP3 and high expressions of LIMD1 and CDC25A may be considered as the molecular signature of the proteins in normal basal/parabasal epithelium. Transmission of the basal- like signature RBSP3 occurred during tumorigenesis, but the signature of LIMD1 and CDC25A was altered and hence not transmitted during development and progression of tumours.

### Expression of RBSP3 and LIMD1 in normal head and neck epithelium and during tumorigenesis was due to inverse promoter methylation

Promoter methylation was performed by MSRA, followed by validation using MSP (Fig 2C and 2D). Promoter methylation frequency of RBSP3 was significantly higher (72.5%, 29/ 40cases) in basal/parabasal layers of normal epithelium compared to spinous layer (17.5%, 7/ 40 cases) (Fig 2E i). Overall methylation frequency in dysplasia was 60%, with comparable frequencies in the mild/ moderate dysplastic lesions (71%) and severe dysplastic lesions (57%) (Fig 2E i). However, early invasive lesions (Stage I) showed low frequency of promoter methylation (31%) with gradual increase with progressing stages II to IV (35–47%) (Fig 2E i).

LIMD1 exhibited comparatively low frequency of methylation (in both basal/parabasal (20%, 8/40) and spinous (15%, 6/ 40) layers of normal epithelium. Interestingly, this trend was opposite to that observed for protein expression. Significantly high methylation was observed in dysplastic lesions (45%, mild/ moderate dysplastic lesion and severe dysplasia) with gradual increase (50–67%) with progression of the tumor (Fig 2E ii). As previously reported, there was absence of methylation of CDC25A throughout carcinogenesis[6].

Significant concordance existed between MSRA and MSP, as determined by Fischer’s exact test (S2 Table). However, similar to previous observations, Groups 1–4 showed no significant difference in promoter methylation of genes during tumorigenesis, annulling effect of etiological factors (Fig 2F).

Thus, the expression signature of RBSP3 and LIMD1 in different layers of normal oral epithelium was maintained through inverse promoter methylation. RBSP3 transmitted its basal- like promoter methylation signature during tumorigenesis, but LIMD1 gradually lost its expression during tumorigenesis through subsequent promoter methylation.

### Deletion of the genes increases from dysplastic lesions to HNSCC

**Fig 4:**
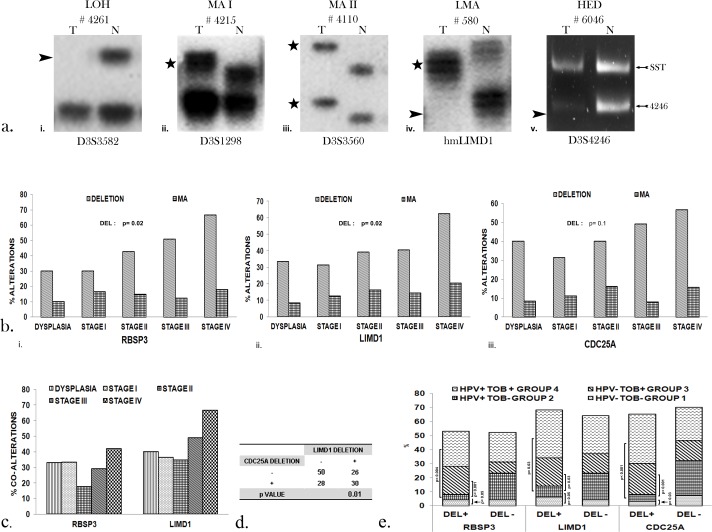
Incidence of genetic deletion. Study of deletion of the genes in dysplasia and HNSCC. a. Representative autoradiograms and agarose gel showing the different types of alterations observed. Type of marker used is given below. LOH: Loss of heterozygosity; MA I: Microsatellite size alteration in one allele; MA II: Microsatellite size alterations in both alleles; LMA: Loss of heterozygosity in one allele and microsatellite size alteration in the other allele. # represents sample number. Arrowhead represents loss of allele; Star alteration change in allele size. b. Histogram representing alterations of the genes in dysplasia and HNSCC. i. RBSP3 ii. LIMD1 iii. CDC25A c. Percentage of co-alterations (deletion+ methylation) of the genes during progression of HNSCC. d. Co-deletion of LIMD1 and CDC25A. e. Histogram representing alterations of the genes in HNSCC in etiological groups I- IV. p value (Fisher’s exact) represents level of significance.

Deletion was performed by microsatellite- based deletion mapping (Fig 4A). Deletion of RBSP3 in dysplastic lesions was high (35%) with comparable frequency in early invasive lesions (stage I/ II) (30–36%), followed by increase in later stages (stage III/ IV) (42–47%) (Fig 4B i).

Deletion frequency of LIMD1 was high (33%) in dysplastic lesions and became comparable in stage I/ II (33–37%) followed by gradual increase in subsequent stages (III/ IV) (47–60%) (Fig 4B ii).Identically, incidence of deletion of CDC25A in dysplasia (40%) was comparable to HNSCC stage I/II (31–41%), with successive increase in stage III/ IV (52–53%) (Fig 4B iii). Deletion of the genes was similar in different grades of HNSCC (data not shown).

Co- alterations (deletion+ methylation) of RBSP3 and LIMD1 were high in the dysplastic lesions (33%, 40% respectively) and HNSCC (29%, 49%) (Fig 4C), with the same increasing with stages of HNSCC, although similar frequencies were observed in Groups 1–4 (data not shown). Additionally, there was significant co- deletion of LIMD1 and CDC25A (p = 0.03, Fig 4D), possibly due to the proximity of their locus at chromosomal region 3p21.31, as reported earlier [6, 7].

Intriguing, although frequency of deletion of the genes was similar in Groups 1, 3 and 4, Group 2 (TOB- HPV+) presented significantly lower deletion compared to the other three groups, indicating that presence of HPV and absence of tobacco might play a pivotal role in preventing genomic instability (Fig 4E).

Additionally, Microsatellite size Alteration (MA) was observed in the following order: LIMD1 (16%) > RBSP3 (12%) > CDC25A (7%) (Fig 4B) with similar distribution in the etiological groups (data not shown).

### Mutation of the genes might not play an important role in HNSCC

No mutations were observed in the different protein domains in samples screened (data not shown). Moreover, encompassing SNPs presented major alleles in all cases. Therefore, mutation may be an infrequent event in the exons screened.

### Etiological factors in conjunction to genetic/ epigenetic alterations were necessary for the development of HNSCC

**Fig 5:**
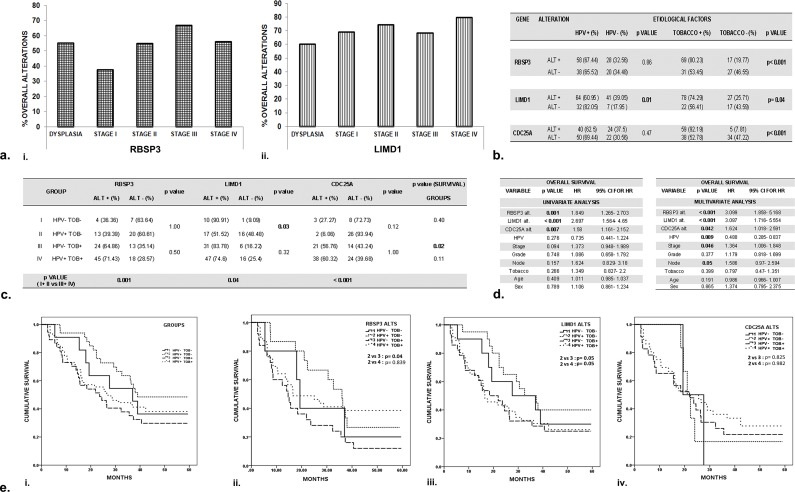
Statistical correlation and analysis of survival of HNSCC patients. a. Overall alterations of the genes in dysplasia and different stages of HNSCC. i. RBSP3; ii. LIMD1 b. Association of alteration (deletion+ methylation) of the candidate genes with etiological factors HPV and tobacco (TOB) in HNSCC. ALT: Alteration. +: Present; -: Absent c. The combined effect of etiological factors HPV and TOB on alterations of the genes in different etiological groups. d. Univariate (by Fischer’s exact test) and Multivariate (by Multivariate Cox proportional hazard regression model) analysis to determine prognostic importance of alterations and different clinic-pathological parameters of patients. HR: Hazard ratio; CI: Confidence interval. e. Kaplan -Meir analysis, followed by Log Rank test to predict 5- year disease free survival in patients with relation to the effect of the two etiological factors HPV and tobacco (groups I-IV). Post- operative overall survival was determined from the date of surgery to the date of last follow up, known recurrence or death (up to 5 years). i. In patients irrespective of alterations. ii. RBSP3 alteration; iii. LIMD1 alteration; iv. CDC25A alteration. p value represents level of significance in different statistical tests. Group 1: HPV-TOB-; Group 2: HPV+TOB-; Group 3: HPV-TOB+; Group 3: HPV+TOB+.

RBSP3 showed high overall alterations (deletion/ methylation) in dysplasia (55%), which although lower in stage I of HNSCC (38%), gradually increased in successive stages (55–67%) (Fig 5A i). In contrast, overall alterations of LIMD1 progressively increased from dysplasia (60%) to enhancing stages of the disease (69–79%) (Fig 5A ii) with unremarkable differences between etiological groups for both genes (data not shown). There was significant concordance between expression and alterations of the genes during tumorigenesis (Tables 2 and 3).

**Table 2:** Overall concordance obtained between different experiments undertaken in dysplastic lesions. Abbreviations: EXPR: Expression (IHC); METH: Methylation (promoter); DEL: Deletion; OVERALL: overall alterations (Deletion+ Methylation);DYS: Dysplasia; HNSCC: Head and neck squamous cell carcinoma; H: High; M: Moderate; L: Low; ND: Not done; NOR: Normal; TUM: Tumor; B: Basal; P: Parabasal; S: Spinous. p value represents level of significance.

	RBSP3: DYSPLASIA				LIMD1: DYSPLASIA				CDC25A: DYSPLASIA		
											
REGN #	ALTERATION			OVER	ALTERATION			OVER	ALTERATION		
	EXPR	METH	DEL	ALL	EXP	METH	DEL	ALL	EXP	METH	DEL
172	L	-	-	-	M	-	-	-	H	-	-
106/13	M	-	-	-	L	+	-	+	M	-	-
15	M	+	ND		H	-	ND		H	-	ND
10	M	+	ND		H	-	ND		H	-	ND
697	H	-	-	-	M	-	-	-	M	-	-
598/13	L	-	-	-	L	+	-	+	M	-	-
8	M	-	+	+	H	+	+	+	M	-	-
620/13	M	+	+	+	M	-	-	-	H	-	-
dc13	L	-	ND		L	+	ND		H	-	ND
acm	L	-	ND		L	+	ND		L	-	ND
dc14	H	+	-	+	H	-	-	-	L	-	+
5	L	-	-	-	M	+	-	+	L	-	+
4	L	+	-	+	M	-	-	-	M	-	-
3	L	-	ND		H	+	ND		M	-	ND
2	H	+	-	+	L	+	+	+	M	-	-
1	H	-	-	-	M	-	+	+	H	-	-
191	M	-	-	-	M	-	-	-	H	-	-
697	M	+	-	+	H	-	ND		M	-	ND
195	L	+	ND		L	+	ND		M	-	ND
181/10	M	-	-	-	H	-	-	-	H	-	-
218/10	H	-	ND		M	-	ND		H	-	ND
258/11	L	+	-	+	L	+	-	+	M	-	+
85/11	M	+	-	+	M	-	-	-	M	-	-
300/11	M	+	ND		H	-	ND		H	-	ND
76/50	M	+	-	+	M	+	-	+	M	-	-
ak	H	-	-	-	H	-	+	+	M	-	-
05/92	M	-	ND		L	+	ND		M	-	ND
01/9r	L	+	ND		H	-	ND		M	-	ND
276/11	H	+	ND		M	-	ND		H	-	ND
283/12	L	+	ND		H	-	ND		M	-	ND
292/12	L	+	ND		M	+	ND		L	-	ND
JE9	M	-	ND		M	-	ND		L	-	ND
263/11	M	+	ND		L	+	ND		L	-	ND
13	H	-	ND		H	-	ND		M	-	ND
DS2	M	-	ND		L	+	ND		M	-	ND
170/10	L	+	ND		H	+	ND		H	-	ND
7	H	-	ND		M	-	ND		M	-	ND
188/10	L	+	ND		L	+	ND		L	-	ND
020/9	L	+	ND		L	+	ND		L	-	ND
17	L	+	ND		M	-	ND		M	-	ND
LE9	M	-	ND		L	+	ND		H	-	ND
196	L	+	ND		M	-	ND		M	-	ND

**Table 3:** Overall concordance obtained between different experiments undertaken in invasion tumours. Abbreviations: EXPR: Expression (IHC); METH: Methylation (promoter); DEL: Deletion; OVERALL: overall alterations (Deletion+ Methylation);DYS: Dysplasia; HNSCC: Head and neck squamous cell carcinoma; H: High; M: Moderate; L: Low; ND: Not done; NOR: Normal; TUM: Tumor; B: Basal; P: Parabasal; S: Spinous. P value represents level of significance.

REG	RBSP3: ALTERATIONS IN HNSCC								LIMD1: ALTERATIONS IN HNSCC								CDC25A: ALTERATIONS IN HNSCC						
NO.	PROTEIN EXP			METH			DEL	OVER	PROTEIN EXP			METH			DEL	OVER	PROTEIN EXP			METH			DEL
	NOR		TUM	NOR		TUM		ALL	NOR		TUM	NOR		TUM		ALL	NOR		TUM	NOR		TU	
	B/ P	S		B/ P	S				B/ P	S		B/ P	S				B/ P	S		B/ P	S		
3962	L	M	M	-	-	-	+	+	H	M	M	-	-	-	-	-	H	M	M	-	-	-	-
4095	L	M	H	+	-	-	-	-	H	M	M	-	-	+	-	+	M	M	M	-	-	-	-
3981	L	H	M	+	-	+	-	+	M	M	L	-	-	+	+	+	M	M	M	-	-	-	-
3422	L	H	M	+	-	-	-	-	L	L	L	+	+	+	+	+	H	H	M	-	-	-	+
3294	M	H	H	-	-	-	-	-	L	L	L	+	-	+	-	+	M	M	L	-	-	-	+
4893	L	M	M	+	+	-	-	-	M	M	L	-	-	+	+	+	M	M	M	-	-	-	-
1941	L	H	L	+	-	+	+	+	H	H	M	-	-	-	-	-	H	H	H	-	-	-	-
3569	L	H	H	+	-	-	-	-	H	H	M	-	-	+	-	+	H	H	M	-	-	-	-
5111	L	H	L	+	-	+	+	+	H	M	L	-	-	-	+	+	L	L	L	-	-	-	-
4567	M	M	M	-	-	+	-	+	M	M	H	-	-	-	-	-	H	M	M	-	-	-	+
1882	L	H	M	+	-	+	-	+	H	M	L	-	-	+	-	+	H	M	H	-	-	-	-
1652	L	H	M	-	-	-	-	-	H	H	L	-	-	-	+	+	L	L	L	-	-	-	+
1976	L	H	L	+	-	-	+	+	H	M	L	-	-	-	-	-	H	M	M	-	-	-	-
2118	L	L	L	+	+	+	-	+	M	M	L	-	-	+	-	+	M	M	M	-	-	-	+
1215	L	M	M	+	+	-	+	+	L	L	H	-	+	-	-	-	M	M	M	-	-	-	-
5852	H	H	L	-	-	+	+	+	H	M	H	-	-	-	-	-	H	H	M	-	-	-	-
3674	L	H	L	+	-	+	-	+	H	M	L	-	-	+	-	+	L	L	L	-	-	-	+
1439	M	H	M	-	-	+	-	+	H	M	L	-	-	+	-	+	H	H	L	-	-	-	+
2117	L	H	L	+	-	+	+	+	L	L	L	+	-	+	-	+	H	H	H	-	-	-	-
1231	L	H	H	+	-	-	-	-	H	M	H	-	-	-	-	-	L	L	L	-	-	-	-
1339	L	L	L	+	+	+	+	+	M	L	M	-	+	-	-	-	H	H	M	-	-	-	-
1226	M	M	M	+	-	-	-	-	M	L	L	-	-	+	+	+	L	L	L	-	-	-	+
1500	M	H	H	-	-	-	-	-	H	M	M	-	-	-	-	-	M	M	H	-	-	-	-
1740	L	M	L	+	+	+	-	+	H	M	M	-	-	-	+	+	M	M	L	-	-	-	+
2036	L	M	L	+	+	-	+	+	L	L	L	+	-	+	-	+	L	L	L	-	-	-	-
1973	L	H	L	+	-	-	+	+	H	H	L	-	-	+	+	+	H	H	M	-	-	-	+
1445	M	H	L	-	-	-	+	+	H	M	L	-	-	+	+	+	H	M	H	-	-	-	-
4663	L	H	M	+	-	-	-	-	L	L	L	+	-	+	+	+	H	M	M	-	-	-	-
2287	M	H	H	+	-	-	-	-	L	L	L	+	+	+	-	+	H	H	M	-	-	-	-
1898	L	H	L	-	-	-	+	+	L	L	M	+	-	+	-	+	L	L	L	-	-	-	+
2209	L	M	M	+	-	-	-	-	H	H	L	-	-	+	+	+	H	H	H	-	-	-	-
3052	L	L	L	+	-	+	+	+	M	M	H	-	-	-	-	-	H	M	H	-	-	-	-
1306	L	H	M	-	+	-	-	-	H	M	L	-	+	+	+	+	H	M	M	-	-	-	+
1812	L	M	L	+	-	+	+	+	H	M	L	-	-	-	+	+	H	H	L	-	-	-	+
2041	L	H	M	+	-	-	+	+	H	M	L	-	-	+	+	+	M	M	M	-	-	-	-
2201	L	M	L	+	-	+	+	+	M	L	L	-	-	+	+	+	M	M	M	-	-	-	-
2670	L	H	H	+	-	-	-	-	L	L	L	+	-	+	+	+	H	M	L	-	-	-	-
1340	H	H	L	-	-	+	-	+	M	M	H	-	+	-	-	-	M	M	L	-	-	-	-
3934	L	L	L	+	-	+	+	+	H	L	M	-	-	-	-	-	H	H	M	-	-	-	-
1545	L	M	L	+	-	-	-	-	H	M	M	-	-	-	-	-	L	M	L	-	-	-	-
2041	L	M	M	ND	ND	-	+	+	M	L	L	ND	ND	+	+	+	M	M	M	-	-	-	-
2118	L	H	M	ND	ND	+	-	+	H	M	M	ND	ND	+	-	+	H	H	L	-	-	-	+
2201	M	M	L	ND	ND	+	+	+	H	M	L	ND	ND	+	+	+	M	M	L	-	-	-	-
3284	L	M	L	ND	ND	-	+	+	H	M	L	ND	ND	-	+	+	M	M	L	-	-	-	+
3636	L	L	L	ND	ND	-	+	+	L	L	L	ND	ND	+	+	+	M	M	L	-	-	-	+
3804	L	H	L	ND	ND	+	-	+	H	M	L	ND	ND	+	+	+	L	M	L	-	-	-	+
3533	L	H	L	ND	ND	-	+	+	M	M	L	ND	ND	+	+	+	H	H	L	-	-	-	+
280	M	M	L	ND	ND	+	+	+	H	H	L	ND	ND	+	+	+	H	H	L	-	-	-	+
1973	L	M	L	ND	ND	+	+	+	H	H	L	ND	ND	-	+	+	M	M	L	-	-	-	+
3798	H	H	L	ND	ND	+	+	+	L	L	M	ND	ND	+	-	+	H	M	L	-	-	-	+
725	L	L	L	ND	ND	-	+	+	M	M	M	ND	ND	-	+	+	M	M	M	-	-	-	-
3131	M	M	H	ND	ND	+	-	+	H	H	L	ND	ND	+	-	+	H	H	L	-	-	-	-
3258	L	M	L	ND	ND	-	+	+	H	M	L	ND	ND	+	+	+	L	L	M	-	-	-	-
1445	L	L	L	ND	ND	-	+	+	H	H	L	ND	ND	+	+	+	H	H	M	-	-	-	-
3758	M	H	M	ND	ND	-	-	-	H	M	M	ND	ND	+	-	+	M	M	L	-	-	-	-
5272	L	M	L	ND	ND	+	+	+	L	L	H	ND	ND	-	-	-	L	M	L	-	-	-	-
4203	L	M	M	ND	ND	+	-	+	H	M	L	ND	ND	+	+	+	M	M	L	-	-	-	+
4480	L	H	H	ND	ND	-	-	-	H	M	M	ND	ND	+	-	+	H	M	L	-	-	-	-
5438	L	H	L	ND	ND	+	+	+	M	L	H	ND	ND	-	-	-	M	M	L	-	-	-	+
1652	L	L	M	ND	ND	-	-	-	M	M	L	ND	ND	-	+	+	L	L	M	-	-	-	+
1268	L	H	L	ND	ND	-	+	+	M	L	L	ND	ND	+	-	+	H	H	L	-	-	-	+
1812	L	M	L	ND	ND	+	+	+	H	M	L	ND	ND	-	+	+	L	L	L	-	-	-	+
1750	L	M	M	ND	ND	-	-	-	M	M	L	ND	ND	-	-	-	M	M	M	-	-	-	-
1231	L	L	M	ND	ND	-	-	-	L	L	M	ND	ND	-	-	-	H	M	M	-	-	-	-

Alterations (deletion/ methylation) in the genes showed significant positive correlation with the habit of tobacco (RBSP3, CDC25A: p< 0.001, LIMD1: p = 0.04). However, for LIMD1, HPV infection was inversely correlated with alterations (p = 0.01) (Fig 5B). In TOB- Group (1, 2), while RBSP3 showed similar frequency of alterations, LIMD1 exhibited significantly diminished alterations in HPV+ patients (Group 2) compared to HPV- Group (1) (p = 0.03). No such disparity was observed between TOB+ groups (3, 4) (Fig 5C). However, there was significantly reduced alterations in TOB- Group (1, 2) compared to TOB+ Group (3, 4) (Fig 5C), indicating the importance of tobacco as a possible factor inducing alterations of the candidate genes.

### Etiological (HPV, Tobacco) and demographical factors significantly affected survival of patients with HNSCC

Alterations of RBSP3, LIMD1 and CDC25A were significantly (p = 0.001, <0.001, 0.007 respectively) correlated to overall survival of HNSCC patients by univariate analysis (Fig 5D). Multivariate analysis indicated significant association with HPV negativity, nodal status and stage as important prognostic factors along with alterations of the genes (Fig 5D).

Interestingly, analysis of patient prognosis showed that survival of Group 2 patients was superior compared to Groups 1, 3 or 4, irrespective of the presence or absence of alterations (Fig 5E i). Similarly, in patients with alterations of RBSP3 and LIMD1, Group 2 patients showed significantly better survival than those belonging to other groups, indicating yet again the role of etiological factors in influencing patient prognosis (Fig 5E ii- iv).

## Discussion

Several studies indicate the importance of stem cells in oral basal/parabasal epithelium for maintaining tissue integrity [3]. Our study thus aims to understand the molecular signature of the cell cycle regulatory proteins RBSP3, LIMD1 and CDC25A in normal basal/parabasal epithelium of H&N followed by analysis of the mode of transmission or alteration of the signature during tumorigenesis.

The molecular signature of the proteins in the normal basal/parabasal epithelium was low nuclear/cytoplasmic expression of RBSP3 and high nuclear with high/ moderate cytoplasmic expressions of LIMD1 and CDC25A.Etiological factors (HPV, tobacco) did not influence the above molecular signature, with the results being comparable to previous reports in H&N epithelium [8, 9] and other tissues [18, 19]. RBSP3, acts as a tumour suppressor phosphatase, that is important for regulating RNA polymerase II transcriptional machinery, attenuating BMP signaling and dephosphorylating pRB to block G1/ S cell cycle transition [20, 21]. RBSP3 shows alteration in different cancers including uveal melanoma, lung cancer, cervical cancer, etc. [20, 22, 23]. Differential expression of RBSP3 as observed by us, might be necessary for cellular proliferation and differentiation. LIMD1 is a multi-functional protein involved in stabilization of pRB-E2F interaction, miRNA silencing, Hippo signaling, etc. [24, 25] and is highly altered in different cancers [26, 27, 28]. Similarly, CDC25A phosphatase is involved in cellular processes such as response to DNA damage, mitotic entry [29, 30] and is also important for tumour growth [31, 32, 33]. In line with their functions and our previous studies [8, 9], the maintenance/ loss of expression signature of the genes during head and neck tumorigenesis indicates their functional importance in this malignancy.

Promoter methylation played an important role in governing the molecular signature of RBSP3 and LIMD1 in normal oral epithelium, as observed by its inverse relationship to protein expression. To the best of our knowledge, reports indicating the reason behind varying expression of the genes in different layers of normal oral epithelium are unavailable. Moreover, the technique of using LCM for separately analyzing promoter methylation in different layers of normal oral epithelium has not been reported, although it was used for RNA expression or epigenetic alterations in other organs [13, 34]. Interestingly, no promoter methylation of the genes was observed in the composite normal epithelium, although its low frequency was previously reported [35]. The observed anomaly might be due to the high sensitivity of LCM for separating different layers of normal epithelium, resulting in enrichment of the otherwise sparsely populated basal/ parabasal cells. This might be the reason why methylation in these layers, even in minor frequencies were detected in our study, which was unreported in previous studies where DNA from the entire epithelium was subjected to promoter methylation.

The normal basal- layer like signature of low expression of RBSP3 was transmitted during development of tumours through promoter methylation in addition to deletion. It appears that development of dysplastic lesions required some selection pressure for maintaining low expression of RBSP3 by additional deletion alongside methylation. However, the basal- like signature of LIMD1 and CDC25A was not transmitted during tumour development. Subsequent and significant promoter methylation (for LIMD1 only) and deletion were necessary for their loss of the expression. Separately assaying alterations in the dysplastic/ non- dysplastic zones of dysplastic epithelium by using LCM is a novel approach, although the results were comparable with previous reports using composite dysplastic tissues of H&N and cervix [6, 7, 16, 18, 35, 36]. This could possibly be due to the high frequency of associated alterations. Moreover, till date, no studies have reported the role alterations of these genes in the same set of H&N samples. Interestingly, analysis of the role of etiological factors in influencing genetic deletion indicated that deletion of the genes in tobacco- HPV+ patients (Group 2) was significantly lower than Groups 1/3/4, possibly due to some effect induced by HPV infection.

Finally, assessing alterations with respect to etiological factors indicated that tobacco induced significant alterations of the candidate genes in HNSCC, whereas in the case of LIMD1, HPV infection played a positive role in preventing alterations. Patients in Group-2 (HPV+TOB-) had further reduced alterations with better 5- year disease free survival, indicating the possible preservative role of HPV in preventing genomic instability and providing a better prognosis, as previously reported by us and other researchers [17, 37–39]. Another alternative might be the evolution of an entirely different subgroup of HPV- infected H&N tumors, which resulted in different frequencies of alterations and prognosis compared to the other groups [40, 41], although further studies are warranted for validating the above.

In conclusion, transmission of normal- like signature could help in categorizing RBSP3 as a separate class of TSG, the maintenance TSG, similar to SnoN [42], which alteration of the basal- like signature categorizes LIMD1 and CDC25A in the classical group of TSGs like WWOX, Smad4, PTEN, etc.[43–45].Etiological factors, especially HPV appears important in H&N tumorigenesis and for providing better patient outcome. Thus, our data also suggests the importance ofLIMD1 and CDC25A in conjunction with HPV for use as diagnostic and prognostic markers of HNSCC, whereas RBSP3 as a prognostic marker only.

## Supporting information

S1 Table List of primers used in the study. Abbreviations: M: Methylated; U: Unmethylated.
(DOCX)

S2 Table Concordance between MSRA and MSP in normal, dysplasia and HNSCC. p (Fisher’s exact) represents level of significance between parameters compared.
(DOCX)

## Abbreviations

HNSCC: Head and neck squamous cell carcinoma
H&N: Head and neck
IHC: Immunohistochemistry
HPV: Human papilloma virus
TOB: Tobacco
MA: Microsatellite size alteration
