# Supplementary material for: Differential transmission of the molecular signature of RBSP3, LIMD1 and CDC25A in basal/ parabasal versus spinous of normal epithelium during head and neck tumorigenesis: A mechanistic study
Source: PLoS One. 2018 Apr 19;13(4):e0195937. doi: 10.1371/journal.pone.0195937 (PMC5909606; doi:10.1371/journal.pone.0195937)
Supplement: S1 Table — Abbreviations: M: Methylated; U: Unmethylated. (DOCX) [file pone.0195937.s001.docx]

**Differential alterations of molecular signature of RBSP3, LIMD1 and CDC25A in normal oral epithelium during oral tumorigenesis**

**Shreya Sarkar ^1^, Neyaz Alam ^2^, Jayanta Chakraborty ^2^, Jaydip Biswas ^2^, Syam Sundar Mandal ^3^, Kabita Chatterjee ^4^, Supratim Ghosh^5^ , Susanta Roychoudhury ^6^ , Tyson Sharp ^7^ and Chinmay Kumar Panda^1^ ***

* Corresponding author

Department of Oncogene Regulation,

Chittaranjan National Cancer Institute,

37, S.P. Mukherjee Road, Kolkata 700026, India.

Phone: 91-33-2474-3922,

Fax: 91-33-2475 7606

E Mail: ckpanda.cnci@gmail.com.

| **PURPOSE** | **GENE** | **PRIMER** | **FORWARD** | **REVERSE** | **SIZE (BP)** |
| --- | --- | --- | --- | --- | --- |
|  |  |  |  |  |  |
| **METHYLATION** | **MSRA** | | | | |
|  | **RBSP3** |  | **5' -ACTCCTCCCCACATCTCTCA -3'** | **5' -CGACACAGGGTGAGTGGAG -3'** | **192** |
|  | **LIMD1** |  | **5'-TAGGCAGGTGGAAGTCTTTA-3'** | **5’-CCAGGTCGTCATACTTATCC-3’** | **201** |
|  | **CDC25A** |  | **5’-GAAGTTGCTTAC TGATTGGTG-3’** | **5’-GTATAAATCCAAACAAACGTG-3’** | **269** |
|  | **K1 (β-3A ADAPTIN)** |  | **5’-TGCCCTCTGGACTGGAACCT-3’** | **5’-CCTGAGCCCAGCCCAAGTC-3’** | **445** |
|  | **K2 (RARβ2)** |  | **5’-AGAGTTTGATGGAGTTGGGT-3’** | **5’-CATTCGGTTTGGGTCAATCC-3’** | **229** |
|  | **MSP** | | | | |
|  | **RBSP3** | **METHYLATED (M)** | **5’-TTTTAATTTTTCGAGTTTTTTTGTC -3’** | **5’-TAAATATATCCCGACCCGAA -3’** | **223** |
|  |  | **UNMETHYLATED (U)** | **5’- TTTTAATTTTTTGAGTTTTTTTGT-3’** | **5’-ATAAATATATCCCAACCCAA -3’** | **226** |
|  |  |  |  |  |  |
|  | **LIMD1** | **METHYLATED (M)** | **5’-TGGGGTTATGTTTTTTACGT-3’** | **5’-CTCCAAACCCAAATCGTC-3’** | **229** |
|  |  | **UNMETHYLATED (U)** | **5’-TGGGGTTATGTTTTTTATGT-3’** | **5’-ACCTCCAAACCCAAATCATC-3’** | **231** |
|  |  |  |  |  |  |
|  | **CDC25A** | **METHYLATED (M)** | **5’- TCGTTATTATCGCGAAAGGTC-3’** | **5’- GAATCGACAAAAAAAACCGAA-3’** | **168** |
|  |  | **UNMETHYLATED (U)** | **5’- TTGTTATTATTGTGAAAGGTTGG-3’** | **5’- CAAATCAACAAAAAAAACCAAA-3’** | **169** |
|  |  |  |  |  |  |
|  |  |  |  |  |  |
| **DELETION** | **RBSP3** | **D3S4237 (NON- INFORMATIVE)** | **5' -AAACCCAGTAAAATGCATGTCC -3'** | **5' -AGCTGAAAGTGAGGATACTCCG -3'** | **145** |
|  |  | **D3S1298** | **5' -AGCTCTCAGTGCCACCCC -3'** | **5' -GAAAAATCCCCTGTGAAGCG -3'** | **194 - 220** |
|  |  |  |  |  |  |
|  | **LIMD1** | **D3S3582** | **5' CGATGTGGCTCTGAACTC -3'** | **5' -AGGGCCTGTTTCCCTAAG -3'** | **220 - 236** |
|  |  | **D3S1358** | **5' -ACTGCAGTCCAATCTGGGT -3'** | **5' -ATGAAATCAACAGAGGCTTG -3'** | **97** |
|  |  | **hmLIMD1** | **5'-TAGGCAGGTGGAAGTCTTTA-3'** | **5’-CCAGGTCGTCATACTTATCC-3’** | **201** |
|  |  |  |  |  |  |
|  | **CDC25A** | **D3S3640** | **5' -GATCGCGTGACATTCC -3'** | **5' -TGCTACTTGCTATTTATCAGACC - 3'** | **133 - 141** |
|  |  | **D3S3560** | **5' -CCTTATGCCCTTTGTCAAGA -3'** | **5' -TGCAGTTATGTATGAGAACATCCT -3'** | **179 - 183** |
|  |  | **D3S4246 (NON- INFORMATIVE)** | **5' -ACCTAGAATGGCAGTATTTCAACC -3'** | **5' -GAAAGGTCTTTGTGGGATAATCC -3'** | **131** |
|  |  |  |  |  |  |
|  | **SST** | **CONTROL** | **5’-AACCAGACGGAGAATGATG-3’** | **5’-GAAAGCTAACAGGATGTGAAA-3’** | **176** |
| **PURPOSE** | **GENE** | **PRIMER** | **FORWARD** | **REVERSE** | **SIZE (BP)** |
|  |  |  |  |  |  |
| **MUTATION** | **RBSP3** | **EXON** |  |  |  |
|  |  | **4** | **5' -CATTATACGCTTGACCAGGC -3'** | **5' -GGAAAAGGGAAAACAAAAAGG -3'** | **286** |
|  |  | **5** | **5' -CATTGGTCTAGGCAGGCTTT -3'** | **5' -ACGGAATCAAAAGGAAGCATACTGTA -3'** | **269** |
|  |  | **6** | **5' -TAGCAATTCTTCCTACAGTGG- 3'** | **5' -GGAACCTGTCACCATAGAAA- 3'** | **307** |
|  |  | **7** | **5' -CATTGTGACACGTCTTTTCC- 3'** | **5' -ATGGAGCACGACATTTTCTT -3'** | **245** |
|  |  | **8** | **5' -AAGAAAGTGCTCAAAGTCCG -3'** | **5' -AGTATCCTCACTTTCAGCTC -3'** | **320** |
|  |  |  |  |  |  |
|  | **LIMD1** | **1.1** | **5'-ACACACACACACGGCACCT-3'** | **5'-AGGTGGATTTTGGCCATCTT-3'** | **217** |
|  |  | **1.2** | **5'-AAATCCACCTCCAGCAGCA-3'** | **5'-GGTATGGCCTGGATCTCT-3'** | **252** |
|  |  | **1.3** | **5'-AGCAGAGATCCAGGCCATA-3'** | **5'-ACCCACTCCCTACACTCAG-3'** | **231** |
|  |  | **1.4** | **5'-AGCATCGGCCTGAGTGTAG-3'** | **5'-GCTCCGTTCTCCAAGTTT-3'** | **257** |
|  |  | **1.5** | **5'-ACTTGGAGAACGGAGCACCA-3'** | **5'-GCAGAACTGGAAAGGTAAGA-3'** | **211** |
|  |  | **1.6** | **5’-TCTTACCTTTCCAGTTCTGC-3’** | **5’-AGGGGACCCTCTTTACAA-3’** | **191** |
|  |  | **1.7** | **5'-CCTGCCTGAGTTATCTTGTAA-3'** | **5'-AACTCCACCAGCCTCTCACT-3'** | **247** |
|  |  |  |  |  |  |
|  | **CDC25A** | **7** | **5’-CCATGAATTTTGTCTTACAGC-3** | **5’-AGGAACACACACACACACAC-3’** | **182** |
|  |  | **10** | **5' -ATTCTCTACCTTTGATTTG -3'** | **5' -AATTACCTTGGAGAAGTC -3'** | **130** |
|  |  | **11** | **5' -GTGTGAATAATATATAGGCT -3'** | **5' -AAAACTAGATTCAAGTACC -3'** | **171** |
|  |  | **12** | **5' -CTAATTTTCTCTCAACAGAT -3'** | **5' -ACTTTAAAACCACAGATCCT -3'** | **177** |
