# Supplementary material for: Differential transmission of the molecular signature of RBSP3, LIMD1 and CDC25A in basal/ parabasal versus spinous of normal epithelium during head and neck tumorigenesis: A mechanistic study
Source: PLoS One. 2018 Apr 19;13(4):e0195937. doi: 10.1371/journal.pone.0195937 (PMC5909606; doi:10.1371/journal.pone.0195937)
Supplement: S2 Table — p (Fisher’s exact) represents level of significance between parameters compared. (DOCX) [file pone.0195937.s002.docx]

**Differential alterations of molecular signature of RBSP3, LIMD1 and CDC25A in normal oral epithelium during oral tumorigenesis**

**Shreya Sarkar ^1^, Neyaz Alam ^2^, Jayanta Chakraborty ^2^, Jaydip Biswas ^2^, Syam Sundar Mandal ^3^, Kabita Chatterjee ^4^, Supratim Ghosh^5^ , Susanta Roychoudhury ^6^ , Tyson Sharp ^7^ and Chinmay Kumar Panda^1^ ***

* Corresponding author

Department of Oncogene Regulation,

Chittaranjan National Cancer Institute,

37, S.P. Mukherjee Road, Kolkata 700026, India.

Phone: 91-33-2474-3922,

Fax: 91-33-2475 7606

E Mail: [ckpanda.cnci@gmail.com](mailto:ckpanda.cnci@gmail.com).

| **PROMOTER METHYLATION** | | | | | | | | | | | | | | | | | |
| --- | --- | --- | --- | --- | --- | --- | --- | --- | --- | --- | --- | --- | --- | --- | --- | --- | --- |
|  |  |  | **LIMD1** | |  | **RBSP3** | |  |  |  |  | **LIMD1** | |  | **RBSP3** | |  |
|  |  | **SAMPLE #** | **MSRA** | **MSP** | **p** | **MSRA** | **MSP** | **p** |  |  | **SAMPLE #** | **MSRA** | **MSP** | **p** | **MSRA** | **MSP** | **p** |
|  |  |  |  |  |  |  |  |  |  |  |  |  |  |  |  |  |  |
| **NORMAL** | **B, P** | 3294 | + | + | **0.003** | - | - | **0.004** |  | **HNSCC** | 1750 | - | - | **<0.001** | + | + | **<0.001** |
|  | **S** |  | - | - |  | - | - |  |  |  | 4261 | + | + |  | - | + |  |
|  | **B, P** | 5111 | - | - |  | + | + |  |  |  | 2242 | + | + |  | - | - |  |
|  | **S** |  | - | - |  | - | - |  |  |  | 7079 | + | + |  | - | - |  |
|  | **B, P** | 1215 | - | + |  | + | + |  |  |  | 580 | - | + |  | - | + |  |
|  | **S** |  | + | + |  | + | + |  |  |  | 1956 | + | - |  | + | + |  |
|  | **B, P** | 1439 | - | - |  | - | - |  |  |  | 3551 | + | + |  | + | + |  |
|  | **S** |  | - | - |  | - | - |  |  |  | 2408 | - | - |  | + | + |  |
|  | **B, P** | 1500 | - | - |  | - | + |  |  |  | 3294 | + | + |  | - | - |  |
|  | **S** |  | - | + |  | - | - |  |  |  | 3131 | + | + |  | + | + |  |
|  | **B, P** | 1740 | - | - |  | - | - |  |  |  | 725 | - | + |  | - | + |  |
|  | **S** |  | - | - |  | + | + |  |  |  | 1268 | + | + |  | - | - |  |
|  | **B, P** | 2670 | + | + |  | + | - |  |  |  | 5316 | + | + |  | + | - |  |
|  | **S** |  | - | - |  | - | - |  |  |  | 4564 | + | + |  | - | - |  |
|  | **B, P** | 4663 | + | + |  | + | + |  |  |  | 699 | - | - |  | - | - |  |
|  | **S** |  | - | - |  | - | - |  |  |  | 32 | - | - |  | + | + |  |
|  | **B, P** | 2209 | - | - |  | + | - |  |  |  | 1215 | - | - |  | - | - |  |
|  | **S** |  | - | - |  | - | - |  |  |  | 2287 | + | + |  | - | + |  |
|  | **B, P** | 1545 | + | + |  | + | + |  |  |  | 2036 | + | + |  | - | - |  |
|  | **S** |  | - | + |  | - | - |  |  |  | 1929 | - | - |  | + | + |  |
|  |  |  |  |  |  |  |  |  |  |  | 1941 | - | - |  | + | + |  |
| **DYSPLASIA** | **DYS** | 598/13 | + | + | **0.003** | + | + | **0.003** |  |  | 2670 | - | - |  | + | + |  |
|  | **NON** |  | - | + |  | - | - |  |  |  | 3636 | + | + |  | - | - |  |
|  | **DYS** | NG | - | - |  | + | + |  |  |  | 5711 | + | + |  | - | - |  |
|  | **NON** |  | - | - |  | - | - |  |  |  | 1440 | + | + |  | - | + |  |
|  | **DYS** | 697 | - | - |  | + | + |  |  |  | 4480 | + | + |  | - | - |  |
|  | **NON** |  | - | - |  | - | + |  |  |  | 1500 | + | + |  | - | - |  |
|  | **DYS** | 218/10 | - | - |  | - | - |  |  |  | 4742 | + | + |  | - | - |  |
|  | **NON** |  | - | - |  | - | - |  |  |  | 3804 | + | + |  | + | + |  |
|  | **DYS** | 300/11 | - | + |  | + | + |  |  |  | 3533 | + | + |  | - | - |  |
|  | **NON** |  | - | - |  | - | - |  |  |  | 5009 | + | + |  | - | - |  |
|  | **DYS** | 8 | + | + |  | + | + |  |  |  | 2117 | + | + |  | + | + |  |
|  | **NON** |  | + | + |  | - | - |  |  |  | 1898 | + | + |  | - | + |  |
|  |  | 01/9R | + | + |  | - | - |  |  |  |  |  |  |  |  |  |  |
|  |  | 85/11 | + | + |  | - | - |  |  |  |  |  |  |  |  |  |  |
